# Utility of Pelvic Magnetic Resonance Imaging in a Younger Population

**DOI:** 10.7759/cureus.116381

**Published:** 2026-09-16

**Authors:** Yuki Ida, Rikiya Sano, Takayuki Komoto, Mae Izumori, Masae Okamoto, Kana Sasaki, Yuko Hara, Ikuo Joja, Takahito Miyake, Tatsuya Miyake

**Affiliations:** 1 Diagnostic Imaging, Miyake Ofuku Clinic, Okayama, JPN; 2 Gynecology, Miyake Ofuku Clinic, Okayama, JPN; 3 Radiology, Miyake Ofuku Clinic, Okayama, JPN; 4 Obstetrics and Gynecology, Miyake Clinic, Okayama, JPN

**Keywords:** adolescent, dysmenorrhea, endometriosis, genital diseases female, magnetic resonance imaging, sexual abstinence

## Abstract

Objective: Pelvic examinations and transvaginal ultrasonography are generally deferred in patients without sexual experience due to potential distress. Transabdominal ultrasonography is widely used but may have variable image quality. Pelvic magnetic resonance imaging (MRI) serves as an alternative to evaluate underlying pathologies. This study investigated the clinical utility of pelvic MRI by comparing clinical presentations and management between adolescents and adults.

Methods: In this single-center, retrospective cohort study, we analyzed 262 female patients without sexual experience (63 adolescents, 10-19 years; 199 adults, ≥20 years) who underwent pelvic MRI between 2024 and 2025. MRI was performed to rule out organic disease. Findings were categorized into organic or functional diseases. Post-imaging therapeutic interventions (hormonal therapy, surgery, symptomatic treatment, and referral) were compared between groups.

Results: Abnormal MRI findings were observed in 87.4% of the cohort. Functional diseases predominated in adolescents (49.2% vs. 34.7%, p<0.001), whereas organic diseases were more prevalent in adults (76.4% vs. 33.3%, p<0.001). The therapeutic intervention rate was significantly higher in adolescents (74.6% vs. 49.2%, p<0.001). Hormonal therapy was the primary treatment in both groups, exceeding 60% in each. However, symptomatic treatment was more frequent in adolescents, whereas surgery was more common in adults (both p<0.05).

Conclusions: Gynecological care for this population must address distinct age-specific clinical profiles. Adolescent organic disease prevalence is higher than expected, with hormonal intervention rates equivalent to adults. In community practice, pelvic MRI effectively guides management to optimize comprehensive women’s health.

## Introduction

Pelvic examinations and transvaginal ultrasonography (US) are essential diagnostic approaches in gynecology. However, due to physical and psychological distress, these examinations are generally deferred in female patients without a history of sexual intercourse. In these cases, transabdominal US is widely used as a primary non-invasive diagnostic modality [1]. It provides essential initial information. Nevertheless, its image quality can vary depending on bladder filling and body habitus [2-4]. These diagnostic factors may influence the timing of condition identification [5] and clinical presentation, potentially impacting long-term symptom management and quality of life [6,7].

Since around 2020, our hospital policy has avoided internal examinations and transrectal US in patients without a history of sexual intercourse. Our outpatient clinic lacks a transabdominal US probe but has an in-house magnetic resonance imaging (MRI) system. Therefore, clinicians naturally chose pelvic MRI for detailed evaluation of underlying pathology. This environment led us to evaluate the usefulness of MRI for this population in daily practice.

MRI offers a valuable complementary approach for clinical decision-making. When pelvic examinations are limited or further clarification is required, MRI can provide objective and comprehensive diagnostic insights [8-10]. This clinical clarity facilitates appropriate individualized care. Options range from conservative strategies to targeted pharmacological management or fertility-sparing surgery [11]. Furthermore, such definitive findings help optimize clinician-patient communication and foster shared decision-making with patients and their guardians.

However, most reports on this population focus on specific diseases or limited age groups. Comprehensive analyses of the disease spectrum across different age groups remain insufficient [12,13]. This study evaluated these groups by comparing adolescents (aged 10-19 years) and adults (aged ≥20 years). Analyzing age-specific clinical profiles and the role of diagnostic imaging holds substantial value. This comparative approach is essential for guiding therapeutic selection, establishing standardized diagnostic workflows, and optimizing distinct, life-stage-specific management for patients without a history of sexual intercourse.

## Materials and methods

This is a single-center, retrospective cohort study analyzing data obtained during routine clinical practice. The study was approved by the Ethics Committee (Approval No. mcg2026-1). Following ethical approval, informed consent was obtained via an opt-out method, allowing patients and their guardians to decline participation.

Eligible patients comprised a consecutive series of 262 women who underwent pelvic MRI for standard diagnostic purposes between January 2024 and December 2025, with electronic medical records confirming no prior history of penetrative sexual intercourse based on medical interview. This cohort included women presenting directly or referred for suspected gynecological symptoms (e.g., refractory dysmenorrhea and abnormal uterine bleeding), health screening abnormalities (e.g., anemia), or follow-up. Except for the follow-up group, patients with confirmed organic disease were excluded. When clinical evaluation indicated the need to rule out underlying organic pathologies, pelvic MRI was performed as an alternative to pelvic examination in patients who requested imaging evaluation.

To evaluate the impact of age, patients were divided into two reference groups: adolescents (aged 10-19 years) and adults (aged ≥20 years). Clinical diagnoses based on pelvic MRI were established through the consensus of two experts, a radiologist and a gynecologist, each with over 15 years of clinical experience.

Organic diseases are characterized by structural alterations in organs. These included uterine fibroids, adenomyosis, endometriosis (endometrioma and deep endometriosis), uterine malformations, ovarian teratomas, ovarian serous cystadenomas, ovarian mucinous cystadenomas, Bartholin gland cysts, uterine prolapse, endometrial polyps, cervical polyps, endometrial thickening, cystic adenomyosis, vaginal wall cysts, paraovarian cysts, peritoneal inclusion cysts, sacral cysts, retroperitoneal cysts, pelvic adhesions, and iliopsoas bursitis. Functional diseases are defined as conditions related to hormone secretion or uterine contraction without structural abnormalities, including functional ovarian cysts, luteal cysts, polycystic ovary (PCO), uterine hypercontractility, uterine hypoplasia, ovarian hypoplasia, constipation, and chronic cystitis. PCO was defined as the presence of 10 or more small follicles in each ovary. Uterine contraction was defined as the loss of the low-intensity junctional zone on multiple planes of T2-weighted images.

We investigated chief complaints, MRI findings, and organic or functional disease patterns for all patients. Treatment strategies were determined by attending gynecologists. Post-MRI interventions were categorized into four groups: maintenance hormonal therapy, surgical intervention, symptomatic treatment, and referral. Symptomatic treatment included analgesics, Kampo (traditional Japanese herbal) medicine as a supportive therapy commonly used in Japan, and hormonal therapy for acute abnormal uterine bleeding. Clinical variables and intervention types were cross-sectionally compared between the two groups.

Statistical analyses were performed using JMP version 14.3.0 (SAS Institute Inc., Cary, NC, USA). Statistical significance was defined as p<0.05. Categorical variables were compared using Pearson’s chi-square test. To identify specific factors contributing to differences in categorical distributions, an adjusted standardized residual analysis was performed, with an absolute residual value greater than 1.96 indicating statistical significance.

## Results

A total of 262 female patients were included in this study. Adolescents (aged 10-19 years) accounted for over 20% of the entire cohort (Figure 1).

**Figure 1:**
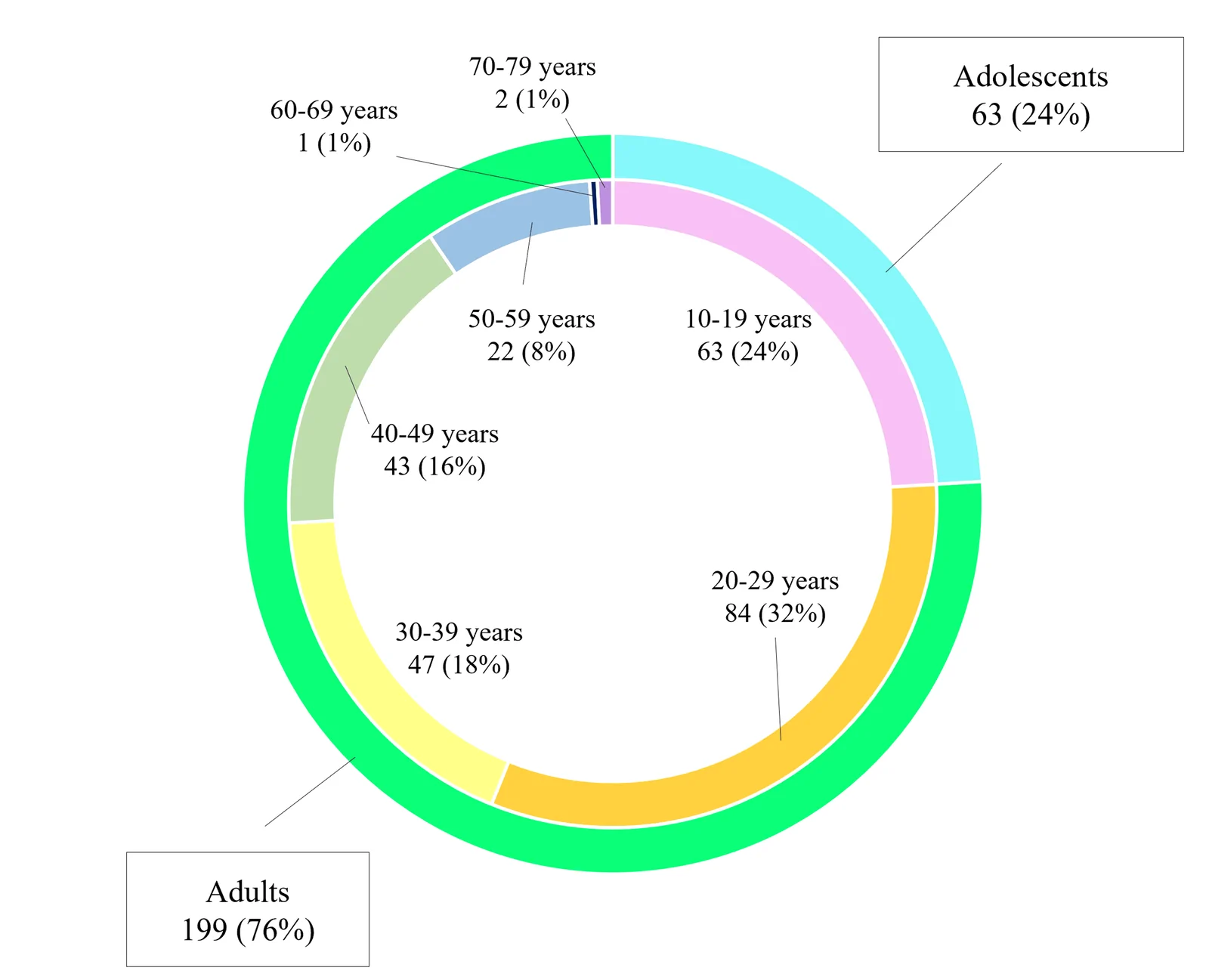
Age distribution of this study population The participants (N=262) are categorized into the adolescent group (aged 10-19 years; n=63, 24.0%) and the adult group (aged ≥20 years; n=199, 76.0%). The breakdown of the adult group by decade is also presented. N: number.

The distribution of the chief complaints is shown in Table 1.

**Table 1:** Clinical diagnoses indicating pelvic MRI: comparison between adolescents and adults Data are given as n (%). AUB: abnormal uterine bleeding; MRI: magnetic resonance imaging; n: number.

Indication for MRI	Adolescents (n=63)	Adults (n=199)	All ages (n=262)
AUB/menstrual disorders	15 (23.8%)	36 (18.1%)	51 (19.5%)
Amenorrhea	5 (7.9%)	3 (1.5%)	8 (3.1%)
Dysmenorrhea	35 (55.6%)	40 (20.1%)	75 (28.6%)
Pelvic pain	2 (3.2%)	11 (5.5%)	13 (5.0%)
Menopausal disorder	0 (0.0%)	1 (0.5%)	1 (0.4%)
Anemia	1 (1.6%)	7 (3.5%)	8 (3.1%)
Urological disorders	0 (0.0%)	1 (0.5%)	1 (0.4%)
Follow-up	3 (4.8%)	87 (43.7%)	90 (34.4%)
Workup	2 (3.2%)	13 (6.5%)	15 (5.7%)
Total	63 (100.0%)	199 (100.0%)	262 (100.0%)

Dysmenorrhea and abnormal uterine bleeding were significantly more prevalent among adolescents than adults. In contrast, adults more frequently underwent MRI for follow-up or workup. Overall, abnormal MRI findings were observed in 87.4% (n=229) of patients (Table 2).

**Table 2:** Comparison of clinical characteristics, imaging findings, and management between adolescent and adult groups Data are given as n (%). Statistical analysis was performed using Pearson's chi-square test. *p<0.05, **p<0.001 indicates a statistically significant difference between the adolescent and adult groups. MRI: magnetic resonance imaging; n: number.

	Adolescents (n=63)	Adults (n=199)	Total (n=262)	Chi-square value	p-value
						Clinical status					
Symptomatic	58 (92.6%)	100 (50.3%)	158 (60.3%)	34.947	<0.001**
MRI assessment					
MRI finding (+)	45 (71.4%)	184 (92.5%)	229 (87.4%)	19.23	<0.001**
Organic disease	21 (33.3%)	152 (76.4%)	173 (66.6%)	39.535	<0.001**
Functional disease	31 (49.2%)	69 (34.7%)	100 (38.2%)	4.282	0.039*
Overlap (organic and functional)	10 (15.9%)	41 (20.6%)	51 (19.5%)	0.683	0.409
						Post-MRI intervention	47 (74.6%)	82 (41.2%)	129 (49.2%)	21.354	<0.001**

Organic diseases were identified in 66.0% (n=173) of patients, and functional diseases in 38.2% (n=100); both conditions coexisted in 19.5% (n=51). Although the abnormal finding rate was significantly higher in adults than in adolescents (p<0.001), over 70% of adolescents exhibited abnormal MRI findings. Specifically, functional and organic diseases were observed in 49.2% (n=31) and 33.3% (n=21) of patients, respectively. The prevalence of organic diseases varied across age groups (Figure 2).

**Figure 2:**
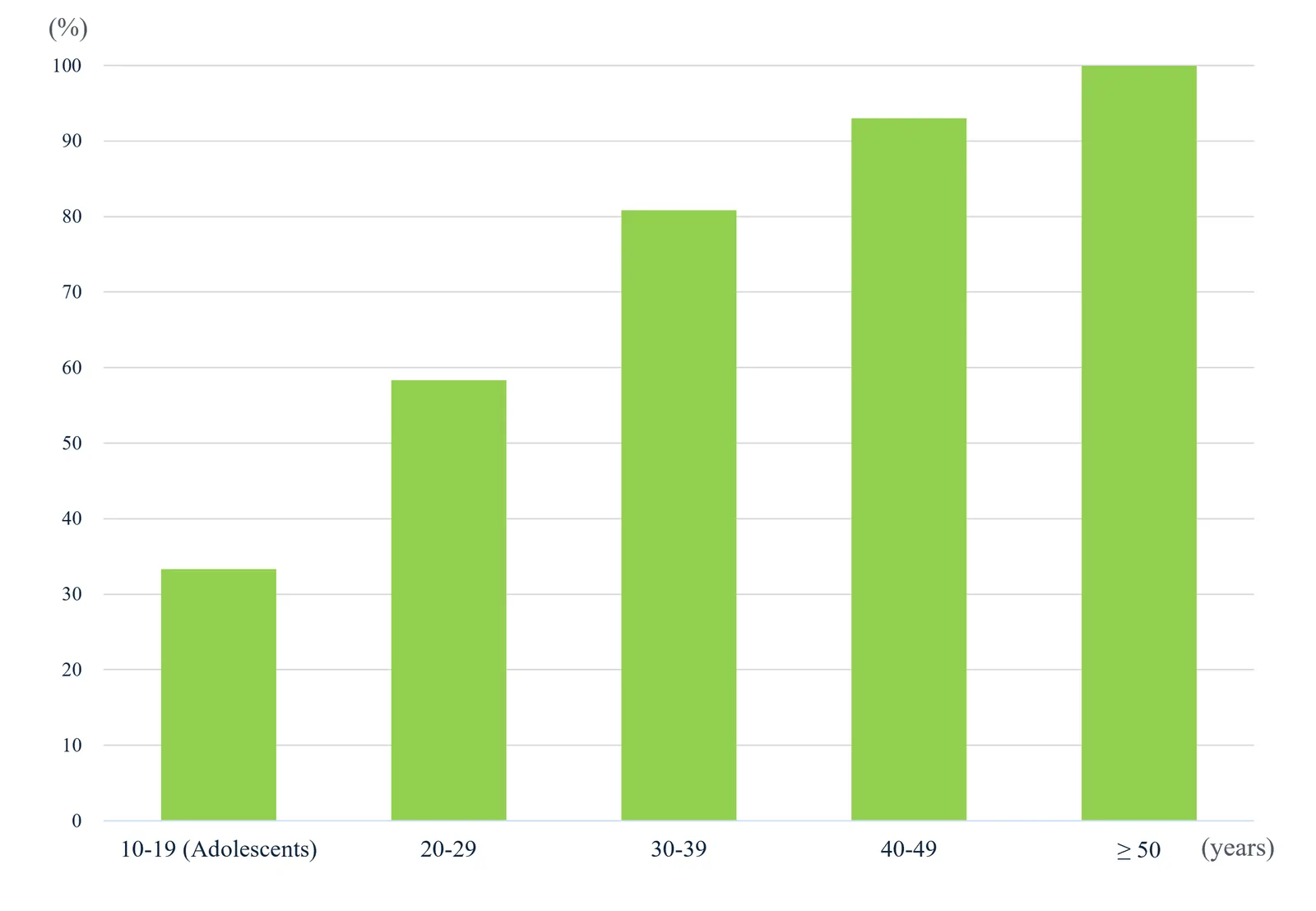
Organic disease detection rates by age group

Intergroup comparisons are summarized in Figure 3.

**Figure 3:**
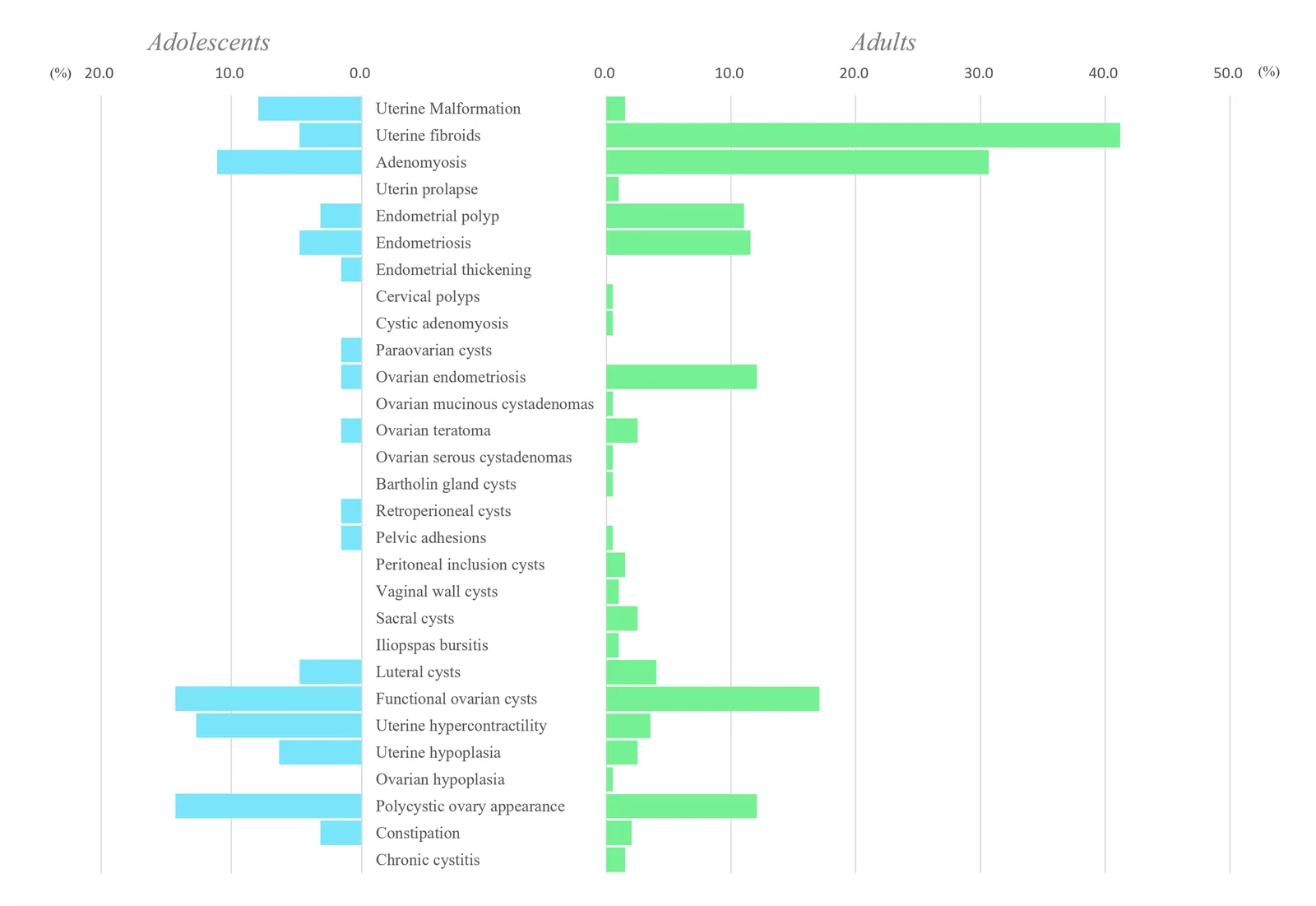
MRI findings by age group Data are presented as percentages of the total number of patients in each group. Individual patients with multiple conditions were counted separately for each category. MRI: magnetic resonance imaging.

Organic diseases were significantly more prevalent among adults (p<0.001), whereas functional diseases were significantly more common among adolescents (p<0.001). The coexistence rate of organic and functional diseases did not differ significantly between groups (≤20% in both groups).

The therapeutic intervention rate following MRI was significantly higher in adolescents than in adults (p<0.001). Treatment was received by 74.6% (n=47) of adolescents. The composition of treatment modalities differed significantly between the groups (p=0.034). Hormonal therapy was the most predominant intervention in both groups, exceeding 60% of all treatments in each. Adjusted standardized residual analysis revealed that symptomatic treatment was significantly more frequent in adolescents (adjusted residual = 2.21, p<0.05), while surgical intervention was significantly more common in adults (adjusted residual = 2.26, p<0.05).

## Discussion

Evidence is lacking regarding whether pelvic MRI offers a diagnostic advantage over pelvic examination or US in patients without a history of sexual intercourse. Direct comparative studies between pelvic MRI and US are limited. In adult infertility, some reported similar diagnostic yield for endometriosis [14], whereas others showed a higher rate for adenomyosis with MRI [15]. In adolescents with severe dysmenorrhea, transabdominal US detects endometriosis lesions in 13.3%-39.7% of cases [16-18]. However, several studies report higher detection rates with MRI, such as unmasking 88% of endometriosis cases missed by US [19] or showing superior sensitivity for peritoneal lesions [20]. Research demonstrating these advantages consistently emphasizes the high objectivity and reproducibility of MRI, which minimize the influence of operator expertise and variable diagnostic criteria.

In our study, pelvic MRI revealed an exceptionally high abnormal rate, including functional condition, across all age groups. This high diagnostic yield suggests that MRI could serve as a reliable, standard diagnostic modality for these patients [1,21]. From a clinical standpoint, integrating MRI into community-based gynecologic clinics provides an effective strategy.

Organic diseases predominated among adults. The higher prevalence of organic diseases among adults likely reflects the cumulative effect of estrogen exposure since menarche [13,22,23]. MRI plays a pivotal role in these cases by precisely identifying the location and extent of lesions, thereby providing objective evidence to confirm the indication for surgery. Conversely, although functional diseases were the primary findings among adolescents, organic abnormalities were still identified in more than 30% of these cases. This high rate underscores the risk of managing adolescents based solely on medical history in the absence of imaging [24,25].

The post-MRI therapeutic intervention rate was significantly higher in adolescents. Importantly, the rate of hormonal therapy in adolescents was equivalent to that in adults. This high rate potentially underscores the limitations of conventional symptomatic therapies [12]. By clarifying the underlying pathology, imaging mitigates ambiguity for clinicians and justifies proactive interventions at an earlier stage.

Emerging evidence suggests that such intractable functional dysmenorrhea in adolescence is a risk factor for future organic conditions, including endometriosis, endometrioma, and adenomyosis [7,26]. Early management not only improves immediate quality of life but may also prevent endometriosis-related infertility [27]. Although higher upfront costs and limited availability remain challenges, clinical utility should be evaluated against long-term outcomes and cost-effectiveness [28]. Early definitive diagnosis can preclude unnecessary follow-up and streamline appropriate treatments [29]. This initial investment may be offset by preventing future chronic pain and infertility treatment.

In managing adolescents, engaging both the patients and their guardians is essential [30]. The visual evidence provided by MRI serves as a critical clinical interface, transforming a subjective symptom into a legitimate target for early medical intervention. Even without apparent organic lesions, this visual clarity enhances motivation, acceptance, and adherence to optimize long-term therapy.

This study has several limitations. First, its retrospective, single-center design may have introduced selection bias. Second, clinical diagnoses were based on pelvic MRI findings rather than histopathological confirmation. Nevertheless, this study possesses unique strengths that address these constraints. Conducting the research at a community-based clinic effectively minimized the referral bias typical of tertiary hospitals to capture a representative disease spectrum of the community population. Furthermore, this study analyzed a large cohort of 262 female patients. These findings provide robust clinical insights essential for the optimization of future diagnostic and management strategies.

## Conclusions

Gynecologic management for patients without a history of sexual intercourse must address distinct clinical characteristics. The prevalence of organic diseases in adolescents is higher than expected. Furthermore, the hormonal intervention rate is equivalent to that observed in adult patients. Within community practice, pelvic MRI offers an effective diagnostic option to guide appropriate management and optimize comprehensive women’s health.
